# Dietary calcium intake and the risk of colorectal cancer: a case control study

**DOI:** 10.1186/s12885-015-1963-9

**Published:** 2015-12-16

**Authors:** Changwoo Han, Aesun Shin, Jeonghee Lee, Jeeyoo Lee, Ji Won Park, Jae Hwan Oh, Jeongseon Kim

**Affiliations:** Department of Preventive Medicine, Seoul National University College of Medicine, 103 Daehakro, Jongno-gu, 110-779 Seoul South Korea; Molecular Epidemiology Branch, Research Institute, National Cancer Center, 323 Ilsan-ro, Ilsandong-gu, Goyang-si, 410-769 Gyeonggi-do South Korea; Center for Colorectal Cancer, National Cancer Center, Goyang-si, Republic of Korea; Department of Surgery, Seoul National University College of Medicine, Seoul, Republic of Korea; Department of Nutritional Science and Food Management, Ewha Womans University, Seoul, Republic of Korea

**Keywords:** Dietary Calcium, Colorectal Cancer, Sub-site Analysis, Case-control study, Korea

## Abstract

**Background:**

High intake of dietary calcium has been thought to be a protective factor against colorectal cancer. To explore the dose-response relationship in the associations between dietary calcium intake and colorectal cancer risk by cancer location, we conducted a case-control study among Korean population, whose dietary calcium intake levels are relatively low.

**Methods:**

The colorectal cancer cases and controls were recruited from the National Cancer Center in Korea between August 2010 and August 2013. Information on dietary calcium intake was assessed using a semi-quantitative food frequency questionnaire and locations of the colorectal cancers were classified as proximal colon cancer, distal colon cancer, and rectal cancer. Binary and polytomous logistic regression models were used to evaluate the association between dietary calcium intake and risk of colorectal cancer.

**Results:**

A total of 922 colorectal cancer cases and 2766 controls were included in the final analysis. Compared with the lowest calcium intake quartile, the highest quartile group showed a significantly reduced risk of colorectal cancer in both men and women. (Odds ratio (OR): 0.16, 95 % confidence interval (CI): 0.11–0.24 for men; OR: 0.16, 95 % CI: 0.09–0.29 for women). Among the highest calcium intake groups, decrease in cancer risk was observed across all sub-sites of colorectum in both men and women.

**Conclusion:**

In conclusion, calcium consumption was inversely related to colorectal cancer risk in Korean population where national average calcium intake level is relatively lower than Western countries. A decreased risk of colorectal cancer by calcium intake was observed in all sub-sites in men and women.

**Electronic supplementary material:**

The online version of this article (doi:10.1186/s12885-015-1963-9) contains supplementary material, which is available to authorized users.

## Background

Diet and nutrition are estimated to explain 30–50 % of the colorectal cancer incidences, which is the third most common cancer in men and the second most common in women worldwide [1, 2]. Evidence from animal studies has suggested that high calcium intake may reduce the risk of colon cancer and recurrence of colorectal adenoma [3]. In addition, a pooled analysis of 10 cohort studies and meta-analyses of observational studies demonstrate the association between high calcium intake and reduced colorectal cancer risk in humans [4–7]. But in randomized clinical trial conducted as a part of the Women’s Health Initiative found no effect of calcium and vitamin D supplementation on colorectal cancer risk and meta-analysis of randomized controlled trials (RCTs) did not show statistically significant effects of calcium supplementation on colorectal cancer risk [8, 9]. Therefore the level of evidence for dietary calcium on colorectal cancer prevention has been considered as “probable” [10].

Many of the previous studies were conducted in the western countries where dietary calcium levels are relatively higher than the Asian countries. Therefore, dose-response relationship in low ranges of calcium intake and risk of colorectal cancer has been inadequately evaluated. In addition, pooled analysis of 10 cohort studies suggested a threshold effect of dietary calcium intake on colorectal cancer risk by showing little further reduction in colorectal cancer risk above 1000 mg/day calcium intake [4]. Because previous calcium supplement trial participants showed high baseline levels of calcium intake over 750 mg/day [6], effects of calcium supplementation on trial group could have been minimized in the RCTs. According to the fifth Korea National Health and Nutrition Examination Survey (KNHANES), mean calcium intake level among Koreans was only 507 mg/day [11]. Therefore, study among Korean population may assess dose-response association of low level dietary calcium intake on the risk of colorectal cancer.

Descriptive epidemiologic studies have led to a hypothesis that proximal and distal colon cancers might have different risk factors [12–15]. Recent reports have demonstrated that proximal and distal colon cancers exhibit different clinical and biological characteristics [16–18]. A previous study in Korea reported that risk factors such as height, family history of cancer, alcohol consumption, and meat consumptions differed by colorectal cancer sub-sites [19]. In addition, few cohort studies conducted on different race and ethnicity did not show consistent association between dietary calcium and colorectal cancer risk by cancer location [20–25]. Although the pathogenesis of these differences by location is unclear, examining colorectal cancer by sub-sites and its association with dietary calcium intake may help to improve the knowledge of proximal, distal, and rectal cancer etiology.

Therefore, in this case-control study, we aimed to explore the dose-response relationship between dietary calcium intake and colorectal cancer risk in the Korean population, where national average calcium intake level is relatively lower than western countries. We also examined whether there are differences in the association between dietary calcium intake and the risk of colorectal cancer by sub-sites of colorectum.

## Methods

### Study population

Eligible colorectal cancer patients were recruited from the Center for Colorectal Cancer, National Cancer Center in Korea from August 2010 to August 2013. Among the 1427 eligible colorectal cancer patients who were hospitalized for an elective cancer surgery, 1259 patients were contacted, and 1070 agreed to participate in the study. Patients who did not complete a structured questionnaire were excluded and total 922 colorectal cancer patients remained in the final analysis. Eligible controls were recruited from participants who visited the Center for Early Detection and Prevention of the National Cancer Center in Korea for a health check-up program from March 2010 to November 2013. The health check-up program is provided bi-annually by the National Health Insurance Cooperation (NHIC), which covers entire Korean population including legal foreign residents, NHIC beneficiaries, and their dependents aged over 40. A total of 5936 participants completed the lifestyle questionnaire and food frequency questionnaire. Participants with implausible calorie intake (<=500 kcal/day or > =4000 kcal/day) were excluded in the analysis. Our initial plan was to match 2766 controls to 922 colorectal cancer patients by age groups and sex. However, due to large number of older cancer patients aged over 60, we were unable to fully match age groups in men. All participants provided written informed consent to participate, and the study protocol was approved by the Institutional review board of the National Cancer Center (IRB No. NCCNCS-10-350).

### Variables

Information on age, marital status, education level, cigarette smoking and alcohol drinking habits, household income, regular exercise and family history of cancer were obtained by a trained interviewer using a structured questionnaire. The locations of colorectal cancer were classified as proximal colon (C18.0–18.4), distal colon (C18.5–18.7), and rectum (C19, C20) by using International Statistical Classification of Diseases and Related Health Problems 10th Revision [26]. Height and weight were measured before surgery for cases and during health examination for controls. Body Mass Index (BMI) was calculated as weight in kilograms divided by height in meters squares and used to define overweight (BMI ≥ 25). Engaging in regular exercise was considered if the participants underwent moderate physical activity at least once a week. Moderate physical activity was defined as “3 or more days of vigorous activity in a week at least 20 min/day” or “5 or more days of moderate-intensity activity and/or walking at least 30 min/day” or “any combination of walking, moderate-intensity or vigorous intensity activities achieving a minimum of 600 MET-minutes/week”. The regular dietary intake of each study participant was estimated by using a semi-quantitative food frequency questionnaire (SQFFQ). The reliability and validity of the food frequency questionnaire was demonstrated in a previous report [27]. The food frequency questionnaire consisted of 106 food items, and participants were asked to report the average frequencies and portion sizes of the foods they ate during the previous year. For each of the 106 food items, nutrient quantity per 100 g was calculated and converted to a daily nutrient intake. Dietary calcium intake was defined as calcium from food, not from supplements and the correlation coefficient between calcium intake from food frequency questionnaire and 12 days dietary records were 0.51–0.54 in the prior validation study [27]. Dietary calcium from dairy foods or non-dairy foods was estimated. Computer-Aided Nutritional analysis Program (CANPro) version 3.0, which is a nutrient database developed by the Korean Nutrition Society, was used to estimate nutrients intakes.

### Statistical analysis

To compare general characteristics between the cases and controls, chi-square tests were performed, and Cochran-Mantel-Haenszel chi-square tests were used to adjust for age. A residual method was used to adjust for individual total energy intake and adjusted dietary calcium intake were classified into sex-specific quartiles [28].

We used regression models to assess the association between daily dietary calcium intake and the risk of colorectal cancer. Age, marital status, educational level, household income, BMI, smoking status, alcohol consumption, regular exercise, and family history of cancer were selected as potential covariates based on literature review [8, 10, 19]. After applying the Cochran-Mantel-Haenszel chi-square test, only significant covariates (*p* < 0.1) that predicted colorectal cancer risk were selected for the regression model after considering multi-colinearity. We also adjusted for dietary fiber intake and calcium supplement use [29]. In the final model, age, education level, regular exercise, fiber intake, calcium supplement use, and total energy intake by residual methods were included in the analysis. Binary and polytomous logistic regression models were used to calculate the odds ratios (OR) and their 95 % confidence intervals (CI) for each quartile of calcium intake, and tests for trend were derived from logistic regression models with a single term representing the medians of each quartile group. For sensitivity analysis, the association was assessed among calcium supplement non-users.

To further visualize the association, we plotted daily dietary calcium intake and the risk of colorectal cancer stratified by sex and sub-sites using estimates from generalized additive models [30–32]. Effective degree of freedom (maximum 10) for the dietary calcium was automatically selected and applied to semi parametric models by mgcv package of R version 3.0.2 (R foundation for Statistical Computing, Vienna, Austria).

All analyses were performed stratifying by sex, and SAS version 9.4 (SAS Institute Inc., Cary, NC) for used for main analyses.

## Results

Basic characteristics and demographic descriptions of the study participants are presented in Table 1. There were 624 men and 298 women in the case group and 1872 men and 894 women in the control group. Among the men, differences in age groups, educational levels, household income, BMI, alcohol consumption, regular exercise, family history of cancer, colorectal cancer, and calcium supplementation use were observed between the colorectal cancer patients and controls. Among the women, differences in marital status, educational level, household income, smoking status, alcohol consumption, regular exercise, family history of cancer, and calcium supplementation use were observed between the colorectal cancer patients and controls. Mean dietary calcium intake among cases and controls was 463.7 and 450.8 mg/day for men and 474.7 and 536.8 mg/day for women, respectively. Top 3 main sources of dietary calcium were kimchi, tofu and milk (Additional file 1: Table S1).

**Table 1:** General characteristics of the study subjects, N (%)

	Men (*n* = 2496)			Women (*n* = 1192)		
	Case	Control	*P*-value^a^	Case	Control	*P*-value
	(*n* = 624)	(*n* = 1872)		(*n* = 298)	(*n* = 894)	
Age group (years)						
−49	128(20.5)	461(24.6)	<0.001	82(27.5)	246(27.5)	>0.999
50–59	226(36.2)	815(43.5)		111(37.3)	333(37.3)	
60+	270(43.3)	596(31.8)		105(35.2)	315(35.2)	
Marital status						
Married	556(89.1)	1671(89.3)	0.262	216(72.5)	697(78.0)	0.028
Single	66(10.6)	167(8.9)		80(26.9)	184(20.6)	
Missing	2(0.3)	34(1.8)		2(0.7)	13(1.5)	
Education level						
Less than middle school	183(29.3)	250(13.4)	<0.001	138(46.3)	146(16.3)	<0.001
High school	265(42.5)	498(26.6)		103(34.6)	365(40.8)	
College or more	176(28.2)	1024(54.7)		57(19.1)	332(37.1)	
Missing	0(0.0)	100(5.3)		0(0.0)	51(5.7)	
Household income (10000 won/month)						
<200	222(35.6)	348(18.6)	<0.001	99(33.2)	212(23.7)	0.016
200–400	252(40.4)	727(38.8)		134(45.0)	313(35.0)	
>400	150(24.0)	588(31.4)		65(21.8)	230(25.7)	
Missing	0(0.0)	209(11.2)		0(0.0)	139(15.6)	
Body mass index (kg/m^2^)						
<25	431(69.1)	1136(60.7)	<0.001	207(69.5)	661(73.9)	0.133
≥25	192(30.8)	736(39.3)		91(30.5)	233(26.1)	
Missing	1(0.2)	0(0.0)		0(0.0)	0(0.0)	
Smoking status						
Non-smoker	145(23.2)	391(20.9)	0.418	264(88.6)	854(95.5)	<0.001
Ex-smoker	302(48.4)	951(50.8)		15(5.0)	21(2.4)	
Current smoker	177(28.4)	530(28.3)		19(6.4)	18(2.0)	
Missing	0(0.0)	0(0.0)		0(0.0)	1(0.1)	
Alcohol consumption						
Non-drinker	107(17.2)	308(16.5)	<0.001	172(57.7)	567(63.4)	0.031
Ex-drinker	103(16.5)	201(10.7)		26(8.7)	44(4.9)	
Current drinker	414(66.4)	1360(72.7)		100(33.5)	282(31.5)	
Missing	0(0.0)	3(0.2)		0(0.0)	1(0.1)	
Regular exercise						
No	388(62.2)	786(42.0)	<0.001	225(75.5)	403(45.1)	<0.001
Yes	236(37.8)	1073(57.3)		73(24.5)	488(54.6)	
Missing	0(0.0)	13(0.7)		0(0.0)	3(0.3)	
Family history of cancer						
No	391(62.7)	1016(54.3)	<0.001	171(57.4)	446(49.9)	0.038
Yes	233(37.3)	846(45.2)		127(42.6)	438(49.0)	
Missing	0(0.0)	10(0.5)		0(0.0)	10(1.1)	
Family history of colorectal cancer						
No	559(89.6)	1768(94.4)	<0.001	277(93.0)	816(91.3)	0.715
Yes	65(10.4)	94(5.0)		21(7.1)	68(7.6)	
Missing	0(0.0)	10(0.5)		0(0.0)	10(1.1)	
Calcium supplement use within 2 years						
No	621(99.5)	1807(96.5)	<0.001	290(97.3)	729(81.5)	<0.001
Yes	3(0.5)	65(3.5)		8(2.7)	165(18.5)	
Total energy intake (kcal/day), Mean (SD)	2210.4(514.4)	1811.4(553.4)	<0.001	1885.9(534.3)	1674.3(604.4)	<0.001
Total calcium intake (mg/day), Mean (SD)	463.7(211.2)	450.8(248.6)	0.207	474.7(248.0)	536.8(335.1)	<0.001

^a^p-values were calculated by the chi-square test or *t*-test

The characteristics of the study subjects were also assessed by dietary calcium intake quartiles (Additional file 1: Table S2). Among the men, high calcium intake groups had higher educational levels, higher household income, and were more likely to engage in regular exercise. Among the women, high calcium intake groups had higher educational levels, were more likely to engage in regular exercise, and more likely to use calcium supplementation.

Table 2 shows the ORs and 95 % CIs of colorectal cancer risk according to dietary calcium intake. High calcium consumption was inversely associated with colorectal cancer risk. Compared with the lowest quartile of calcium intake (<335 mg/day), the multivariate odds ratio for colorectal cancer in the highest quartile of calcium intake (≥567 mg/day) was 0.16 (95 % CI: 0.11–0.24) in men. In women, the multivariate odds ratio for colorectal cancer was 0.16 (95 % CI: 0.09–0.29) for the highest quartile of calcium intake (≥663 mg/day) compared with the lowest quartile of calcium intake (<380 mg/day).

**Table 2:** Odds ratios (OR) and 95 % confidence intervals (CI) for the association of dietary calcium intake and colorectal cancer risk

	Male (*n* = 2496)				Female (*n* = 1192)		
	Controls/ cases(n)	Age-adjusted OR(95 % CI)	Multivariate OR^a^(95 % CI)		Controls/cases(n)	Age-adjusted OR(95 % CI)	Multivariate OR(95 % CI)
Calcium intake (mg/day)				Calcium intake (mg/day)			
Q1 (<335)	468/202	1.00	1.00	Q1 (<380)	224/125	1.00	1.00
Q2 (335 - < 432)	468/222	1.09(0.86–1.37)	0.92(0.71–1.19)	Q2 (380 - < 519)	223/111	0.89(0.65–1.23)	0.93(0.65–1.34)
Q3 (432 - < 567)	468/141	0.68(0.52–0.87)	0.51(0.38–0.68)	Q3 (519 - < 663)	223/43	0.34(0.23–0.51)	0.39(0.25–0.61)
Q4 (≥567)	468/59	0.28(0.20–0.38)	0.16(0.11–0.24)	Q4 (≥663)	224/19	0.15(0.09–0.25)	0.16(0.09–0.29)
P-value for trend^b^		<0.001	<0.001	P-value for trend		<0.001	<0.001
Dairy food calcium (mg/day)				Dairy food calcium (mg/day)			
Q1 (<11)	468/228	1.00	1.00	Q1 (<20)	223/109	1.00	1.00
Q2 (11 - < 47)	468/222	0.98(0.78–1.23)	1.02(0.80–1.30)	Q2 (20 - < 78)	224/102	0.93(0.67–1.29)	1.01(0.70–1.47)
Q3 (47 - < 146)	468/125	0.55(0.43–0.71)	0.65(0.49–0.85)	Q3 (78 - < 225)	224/64	0.59(0.41–0.84)	0.67(0.45–1.01)
Q4 (≥146)	468/49	0.21(0.15–0.29)	0.28(0.19–0.40)	Q4 (≥225)	223/23	0.21(0.13–0.34)	0.20(0.12–0.35)
P-value for trend		<.001	<.001	P-value for trend		<0.001	<0.001
Non-Dairy food calcium (mg/day)				Non-Dairy food calcium (mg/day)			
Q1 (<279)	468/168	1.00	1.00	Q1 (<302)	224/111	1.00	1.00
Q2 (279 - < 360)	468/230	1.33(1.04–1.68)	1.07(0.82–1.40)	Q2 (302 - < 397)	223/109	0.98(0.71–1.35)	0.89(0.61–1.29)
Q3 (360 - < 470)	468/159	0.91(0.70–1.17)	0.59(0.44–0.80)	Q3 (397 - < 522)	223/58	0.52(0.36–0.75)	0.53(0.35–0.82)
Q4 (≥470)	468/66	0.37(0.27–0.50)	0.16(0.11–0.25)	Q4 (≥522)	224/20	0.18(0.11–0.30)	0.15(0.08–0.27)
P-value for trend		<0.001	<0.001	P-value for trend		<0.001	<0.001

^a^Adjusted by age, education level, regular exercise, fiber intake, calcium supplement use, and total energy intake

^b^Test for trend calculated with the median intake for each category of dietary calcium intake as a continuous variable

In analysis considering sources of dietary calcium, both dairy and non-dairy food calcium showed negative association with risk of colorectal cancer. The highest dairy food calcium intake group showed a reduced risk of colorectal cancer in both men and women (OR: 0.28, 95 % CI: 0.19–0.40 for men; OR: 0.20, 95 % CI: 0.12–0.35 for women). Similarly, the highest non-dairy food calcium intake group showed a reduced risk of colorectal cancer (OR: 0.16, 95 % CI: 0.11–0.25 for men; OR: 0.15, 95 % CI: 0.08–0.27 for women).

The odds ratios for colorectal cancer by sub-sites, according to dietary calcium intake are shown in Table 3. An inverse association between calcium intake and colorectal cancer risk persisted across all sub-sites of colorectum. The odds ratios for colorectal cancer for men in the highest quartile were 0.35 (95 % CI: 0.17–0.74) for the proximal colon cancer, 0.13 (95 % CI: 0.07–0.26) for the distal colon cancer, and 0.13 (95 % CI: 0.08–0.23) for the rectal cancer compared with those in the lowest quartile. By comparing odds ratio of highest quartile across the sub-sites, prominent differences between proximal colon and other sub-sites were observed. In case of women, the odds ratios for colorectal cancer in the highest quartile were 0.13 (95 % CI: 0.03–0.48) for the proximal colon cancer, 0.12 (95 % CI: 0.05–0.32) for the distal colon cancer, and 0.20 (95 % CI: 0.09–0.47) for the rectal cancer compared with those in the lowest quartile.

**Table 3:** Odds ratios (OR) and 95 % confidence intervals (CI) for the association of dietary calcium intake and colorectal cancer sub-sites

	Control	Proximal colon			Distal colon			Rectum		
Total energy adjusted dietary calcium intake (mg/day)	No	No	Age-adjusted OR(95 % CI)	Multivariate OR^a^(95 % CI)	No	Age-adjusted OR(95 % CI)	Multivariate OR(95 % CI)	No	Age-adjusted OR(95 % CI)	Multivariate OR(95 % CI)
Men	*N* = 1872	*N* = 113			*N* = 178			*N* = 320		
Q1 (<335)	468	26	1.00	1.00	60	1.00	1.00	109	1.00	1.00
Q2 (335 - < 432)	468	43	1.63(0.99–2.70)	1.40(0.83–2.37)	63	1.04(0.71–1.51)	0.90(0.60–1.34)	112	1.02(0.76–1.36)	0.85(0.61–1.17)
Q3 (432 - < 567)	468	28	1.04(0.60–1.80)	0.81(0.45–1.45)	41	0.66(0.43–1.00)	0.51(0.33–0.81)	71	0.63(0.45–0.87)	0.47(0.32–0.67)
Q4 (≥567)	468	16	0.58(0.31–1.10)	0.35(0.17–0.74)	14	0.22(0.12–0.40)	0.13(0.07–0.26)^c^	28	0.25(0.16–0.38)	0.13(0.08–0.23)^c^
P-value for trend^b^			0.021	<0.001		<0.001	<0.001		<0.001	<0.001
Women	*N* = 894	*N* = 53			*N* = 113			*N* = 124		
Q1 (<380)	224	26	1.00	1.00	43	1.00	1.00	51	1.00	1.00
Q2 (380 - < 519)	223	18	0.70(0.37–1.32)	0.75(0.39–1.46)	48	1.12(0.72–1.76)	1.11(0.68–1.81)	43	0.85(0.54–1.33)	0.93(0.58–1.52)
Q3 (519 - < 663)	223	6	0.23(0.09–0.57)	0.27(0.11–0.72)	15	0.35(0.19–0.65)	0.35(0.18–0.68)	22	0.43(0.25–0.74)	0.55(0.31–0.99)
Q4 (≥663)	224	3	0.11(0.03–0.38)	0.13(0.03–0.48)	7	0.16(0.07–0.37)	0.12(0.05–0.32)	8	0.16(0.07–0.34)	0.20(0.09–0.47)
P-value for trend			<0.001	0.001		<0.001	<0.001		<0.001	<0.001

^a^Adjusted by age, education level, regular exercise, fiber intake, calcium supplement use, and total energy intake

^b^Test for trend calculated with the median intake for each category of dietary calcium intake as a continuous variable

^c^The odds ratio was statistically different from that of proximal colon (*p* = 0.05 for distal colon and *p* = 0.03 for rectum)

**Fig. 1:**
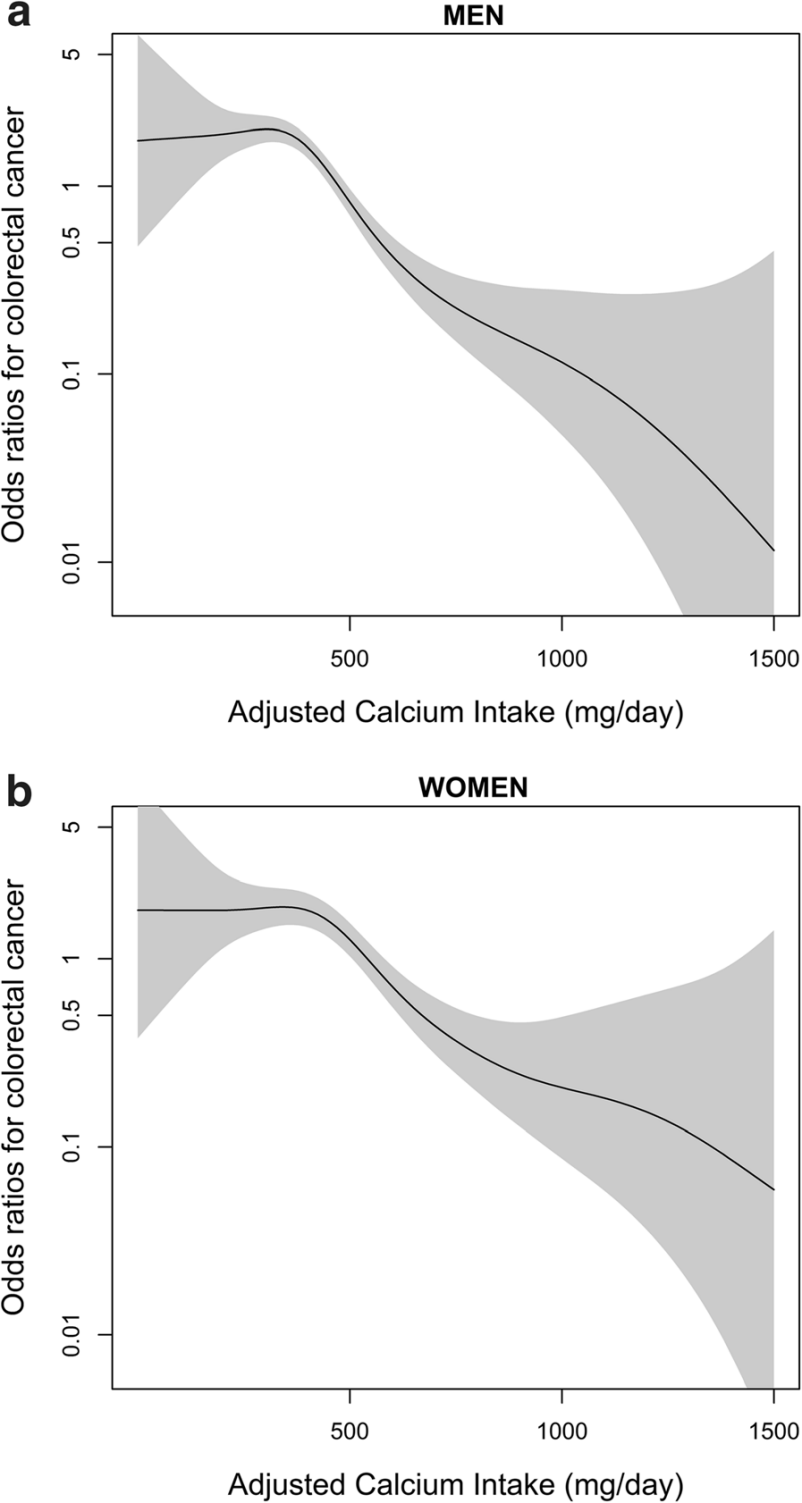
Relationship between total energy adjusted daily calcium intake (mg/day) and colorectal cancer risk. **a** Men. **b** Women. Each figure shows the spline curve (solid line) with a 95 % CI (shaded). The curves are adjusted for age, education level, regular exercise, fiber intake, calcium supplement use, and total energy intake

**Fig. 2:**
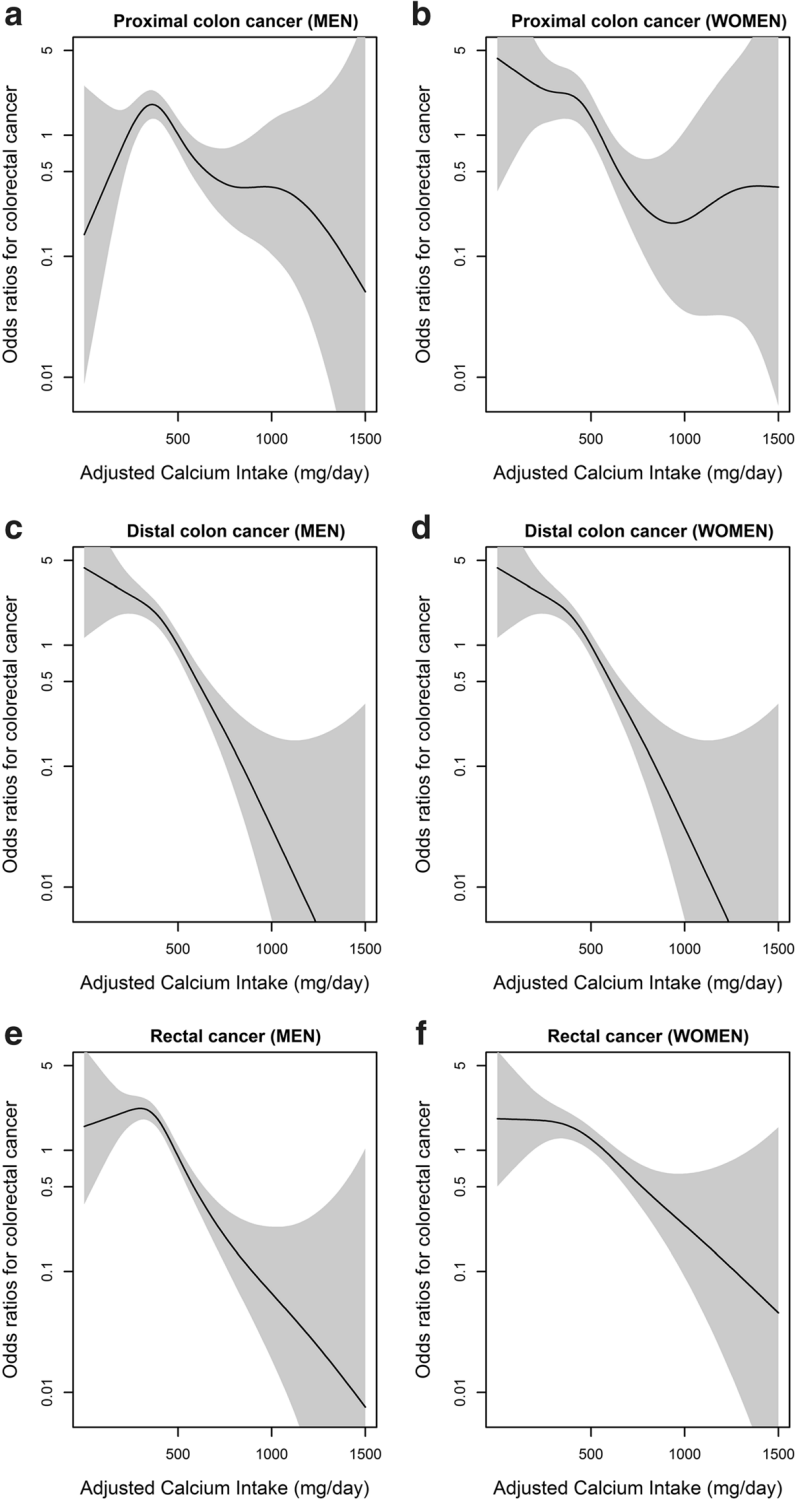
Relationship between total energy adjusted daily calcium intake (mg/day) and colorectal cancer risk in men and women stratified by cancer sub-sites. **a** Proximal colon cancer (Men). **b** Proximal colon cancer (Women). **c** Distal colon cancer (Men). **d** Distal colon cancer (Women), **e** Rectal cancer (Men). **f** Rectal cancer (Women). Each figure shows the spline curve (solid line) with a 95 % CI (shaded). The curves are adjusted for age, education level, regular exercise, fiber intake, calcium supplement use, and total energy intake

Figure 1 shows the relationship between daily dietary calcium intake and the risk of colorectal cancer in men and women. Figure 2 shows the relationship between dietary calcium intake and risk of colorectal cancer regarding colorectal cancer sub-sites in men and women. The Fig. 1 presents distinct patterns of colorectal cancer risk in relation to calcium intake; for example, the overall non-linear relationship between men and women was similar, showing a rapid decrease in the risk of colorectal cancer at calcium intake levels over 500 mg/day and little further reduction in the risk when daily calcium intake exceeded more than 1000 mg/day. (Test for non-linearity, men: p-value <0.01; women: p-value <0.01) The overall spline analyses were concordant with the results from quartile analysis in Tables 2 and 3.

## Discussions

In this case control study, we evaluated the dose-response association between dietary calcium intake and risk of colorectal cancer. Compared with the lowest calcium intake quartile, the highest calcium intake quartile showed significantly reduced risk of colorectal cancer in both men and women. By applying a generalized additive model, both men and women showed a similar non-linear relationship between dietary calcium intake and the risk of colorectal cancer. When dietary sources were considered, calcium intake from both dairy and non-dairy food showed significant negative association with colorectal cancer risk.

According to the KNHANES data, the average daily calcium intake of Korean men and women was 561.0 and 452.6 mg, respectively, which was approximately 70 % of the Korean recommended daily calcium allowance [11]. Daily calcium intake was inadequate in all age groups except for the infant period, and age groups over 65 only consumed 60 % of their recommended daily allowance [11]. If there is a causal relationship between increased calcium intake and decreased colorectal cancer risk, colorectal cancer risk of Korean population may reduce by increasing the amounts of daily calcium consumption up to the recommended level (700–750 mg/day).

There are biologically plausible mechanisms between dietary calcium intake and reduced colorectal cancer risks. Calcium plays protective role against inflammation and bile acid irritation on colonic wall. Intracellular calcium in colonic epithelial cells may reduce cancer promoting inflammatory responses [33], and the presence of ionized calcium may inhibit the toxic and potential irritating effects of fatty acids and free bile acid in the colon [34].

The sub-sites of the colon (proximal and distal) and rectum differs in embryonic origin, morphologic appearance, histologic features, and physiological functions [13, 35, 36]. Embryonically, proximal colon originates from midgut whereas distal and rectum originates from hindgut [36]. Main functions of proximal and distal colons are nutrient and water absorption, and that of rectum is fecal storage before defecation [36]. Wall of the rectum is thicker than colon, and proximal colon has more complex capillary network compared with distal colon and rectum [37, 38]. Distal colon possess high proportion of goblet cells and rectum has high proportion of endocrine cells [39, 40]. Therefore due to various differences between colorectal sub-sites, dietary calcium effects on colorectal cancer risk may differ by cancer location. However, in our study, the association between colorectal cancer risk and daily calcium intake did not vary significantly by colorectal sub-sites. Only statistically significant differences were observed in the highest calcium intake group of men, showing more prominent cancer risk reduction in distal colon and rectum compared with proximal colon.

Few human studies have examined the association between calcium and cancer risk by sub-sites of the colorectum, but the results have been conflicting. In cohort study of Swedish men, multivariate rate ratio (RR) of colorectal cancer risk in the highest quartile calcium intake group (> = 1445 mg/day) was 0.68 (95 % CI: 0.51 to 0.91) compared to lowest quartile calcium intake group (<956 mg/day). In sub-site analysis, proximal colon and rectum showed a significant decrease in cancer risk with high intake of dietary calcium [20]. In cohort study conducted in the United States, the highest quintile calcium intake group (> = 1255 mg/day) showed marginally reduced risk of colorectal cancer in men and women compared to the lowest quintile calcium intake group (<561 mg/day) (RR: 0.87, 95 % CI: 0.67–1.12) [21]. In sub-site analysis, only proximal colon showed marginally reduced risk (RR: 0.57, 95 % CI: 0.28–1.13) of colorectal cancer among the highest quintile calcium intake group in men. In cohort study of women, the highest quintile intake group (> = 830.9 mg/day) showed statistically significant reduction of colorectal cancer risk compared to the lowest calcium intake group (<412.3 mg/day) (RR: 0.74, 95 % CI: 0.56–0.98) [22], and the risk reduction was only observed for proximal colon in sub-site analysis (RR: 0.60, 95 % CI: 0.38–0.97). In Swedish mammography cohort, women aged over 55 with the highest calcium intake (> = 816 mg/day) showed decreased colorectal cancer risk compared to the lowest quartile intake group (<568 mg/day) for overall colorectal cancer (RR: 0.66, 95 % CI: 0.49–0.89) [23], and distal colon cancer (RR: 0.33, 95 % CI: 0.16–0.67). In addition, although total calcium intake was inversely associated with distal colon cancer in pooled analysis of two cohort studies [24], there was no significant association between dietary calcium and colorectal sub-sites in Japanese cohort study [25]. Compared to previous studies, our study participant’s daily calcium intake levels are relatively low. Because marked reduction of colorectal cancer risk in all sub-sites of colorectum has been showed in our study results, dose-response relationship in lower ranges of calcium intake could be suggested from our study.

There are several strengths of our study. First, the association between dietary calcium intake and colorectal cancer risks are analyzed among Korean population, whose average calcium intake is relatively lower than western population. Therefore, assessment of dose-response relationship within low level dietary calcium on risk of colorectal cancer could be made with our analyses. Second, not only the dose-response relationship but also potential differences in risk among sub-sites of colorectum could be assessed in our study. By using graphical methods, we compared patterns of colorectal cancer risk according to dietary calcium intake by each colorectal sub-sites.

Our main limitation comes from the study design and the use of the food frequency questionnaire. First, the controls were recruited from the participants of the health check-up program provided by the National Health Insurance Corporation; therefore, they could have a healthier lifestyle than the colorectal cancer cases. However, since the cases and controls were recruited in the same hospital, characteristics between two groups would be comparable. Second, recall bias is inevitable due to case control study design assessing for prior personal information. However, since dietary calcium intake was estimated from diverse food sources, it is hard to speculate that the cases or controls systematically under- or over reported their calcium intake levels. Third, due to food frequency questionnaire use in our study, potential measurement errors could have affected our study results. The non-differential measurement error, however, would lead the results toward the null values. Fourth, although we asked the study participants to report their food consuming patterns before cancer diagnosis, direct causal inference between dietary calcium consumption and risk reduction of colorectal cancer cannot be made in case-control study design. Fifth, vitamin D intake, which could be a potential confounder, could not be estimated from the nutrients database we used. Lastly, although information on calcium supplementation use during last 2 years were available, we did not ask the dose of calcium supplement that participants consumed. Therefore total calcium intake could not be estimated. However, in sensitivity analysis conducted for participants who did not consume calcium supplements, the association was very similar to the main analysis (Additional file 1: Table S3). In addition, since the proportion of supplement consumers was higher in controls than in cases, the analysis of total intake of calcium would reinforce our findings.

## Conclusions

In conclusion, calcium consumption was inversely related to colorectal cancer risk in Korean population where national average calcium intake level is relatively lower than western countries. A decreased risk of colorectal cancer by calcium intake was observed in all sub-sites of men and women.

## Additional file

Additional file 1:
**Table S1.** Top 10 calcium contributing foods of study population (mg/day). **Table S2.** Characteristics of study subjects according to quartile of energy-adjusted dietary calcium intake N(%). **Table S3.** Odds ratios (OR) and 95% confidence intervals (CI) for the association of dietary calcium intake and colorectal cancer risk among the calcium supplement non-users. (DOC 129 kb)
