# Supplementary material for: Dietary calcium intake and the risk of colorectal cancer: a case control study
Source: BMC Cancer. 2015 Dec 16;15:966. doi: 10.1186/s12885-015-1963-9 (PMC4682267; doi:10.1186/s12885-015-1963-9)
Supplement: Additional file 1: — Table S1. Top 10 calcium contributing foods of study population (mg/day). Table S2. Characteristics of study subjects according to quartile of energy-adjusted dietary calcium intake N(%). Table S3. Odds ratios (OR) and 95% confidence intervals (CI) for the association of dietary calcium intake and colorectal cancer risk among the calcium supplement non-users. (DOC 129 kb) [file 12885_2015_1963_MOESM1_ESM.doc]

Additional file 1: Table S1. Top 10 calcium contributing foods of study population (mg/day)

|  | Case (n=922) | Control (n=2,766) |
| --- | --- | --- |
|  | Mean±SD | Mean±SD |
| Kimchi | 72.85 ± 44.88 | 61.07 ± 47.13 |
| Tofu | 62.93 ± 42.91 | 58.58 ± 56.51 |
| Milk | 51.57 ± 101.44 | 66.88 ± 113.39 |
| Anchovy | 46.96 ± 49.59 | 49.34 ± 72.02 |
| Rice | 18.16 ± 6.51 | 14.22 ± 6.02 |
| White radish | 14.35 ± 23.76 | 22.86 ± 39.21 |
| Yogurt | 12.64 ± 21.38 | 13.97 ± 26.38 |
| Ice cream | 10.17 ± 17.21 | 8.33 ± 18.79 |
| Rice drink | 8.69 ± 16.54 | 12.49 ± 25.20 |
| Coffee cream | 7.15 ± 6.84 | 6.73 ± 6.09 |

Additional file 1: Table S2. Characteristics of study subjects according to quartile of energy-adjusted dietary calcium intake N(%)

|  | Male (n=2,496) | | | | | Female (n=1,192) | | | | |
| --- | --- | --- | --- | --- | --- | --- | --- | --- | --- | --- |
|  | Lowest  (<335) | 2^nd^  (335-<432) | 3^rd^  (432-<567) | Highest  (≥567) | *P-value^a)^* | Lowest  (<380) | 2^nd^  (380-<519) | 3^rd^  (519-<663) | Highest  (≥663) | *P-value* |
| Age group (years) |  |  |  |  |  |  |  |  |  |  |
| -49 | 177(26.4) | 167(24.2) | 135(22.2) | 110(20.9) | 0.211 | 105(30.1) | 104(31.1) | 67(25.2) | 52(21.4) | 0.151 |
| 50-59 | 280(41.8) | 291(42.2) | 251(41.2) | 219(41.6) |  | 124(35.5) | 124(37.1) | 100(37.6) | 96(39.5) |  |
| 60+ | 213(31.8) | 232(33.6) | 223(36.6) | 198(37.6) |  | 120(34.4) | 106(31.7) | 99(37.2) | 95(39.1) |  |
| Marital status |  |  |  |  |  |  |  |  |  |  |
| Married | 599(89.4) | 607(88.0) | 547(89.8) | 474(90.0) | 0.836 | 266(76.2) | 249(74.6) | 208(78.2) | 190(78.2) | 0.507 |
| Single | 64( 9.6) | 69(10.0) | 54( 8.9) | 46( 8.7) |  | 80(22.9) | 82(24.6) | 53(19.9) | 56(20.2) |  |
| Missing | 7( 1.0) | 14( 2.0) | 8( 1.3) | 7( 1.3) |  | 3( 0.9) | 3( 0.9) | 5( 1.9) | 4( 1.7) |  |
| Education level |  |  |  |  |  |  |  |  |  |  |
| Under middle school | 135(20.2) | 132(19.1) | 109(17.9) | 57(10.8) | <0.001 | 96(27.5) | 92(27.5) | 53(19.9) | 43(17.7) | 0.001 |
| High school | 237(35.4) | 208(30.1) | 169(27.8) | 149(28.3) |  | 152(43.6) | 117(35.0) | 102(38.4) | 97(39.9) |  |
| College or more | 277(41.3) | 321(46.5) | 304(50.0) | 298(56.6) |  | 87(24.9) | 113(33.8) | 100(37.6) | 89(36.6) |  |
| Missing | 21( 3.1) | 29( 4.2) | 27( 4.4) | 23( 4.4) |  | 14( 4.0) | 12( 3.6) | 11( 4.1) | 14( 5.8) |  |
| Household income (1,000won/month) | | |  |  |  |  |  |  |  |  |
| <200 | 174(26.0) | 159(23.0) | 144(23.7) | 93(17.7) | 0.003 | 102(29.2) | 87(26.1) | 65(24.4) | 57(23.5) | 0.107 |
| 200-400 | 278(41.5) | 275(39.9) | 218(35.8) | 208(39.5) |  | 143(41.0) | 124(37.1) | 88(33.1) | 92(37.9) |  |
| >400 | 165(24.6) | 207(30.0) | 194(31.9) | 174(32.6) |  | 68(19.5) | 96(28.7) | 73(27.4) | 58(23.9) |  |
| Missing | 53( 7.9) | 49( 7.1) | 53( 8.7) | 54( 10.3) |  | 35(10.3) | 27( 8.1) | 40(15.0) | 36(14.8) |  |
| Body mass index (kg/m^2^) |  |  |  |  |  |  |  |  |  |  |
| <25 | 429(64.9) | 428(62.0) | 391(64.2) | 319(60.5) | 0.523 | 251(71.9) | 242(72.5) | 189(71.1) | 186(76.5) | 0.515 |
| ≥25 | 241(36.0) | 261(37.8) | 218(35.8) | 208(39.5) |  | 98(28.1) | 92(27.5) | 77(29.0) | 57(23.5) |  |
| Missing | 0( 0.0) | 1( 0.1) | 0( 0.0) | 0( 0.0) |  | 0( 0.0) | 0( 0.0) | 0( 0.0) | 0( 0.0) |  |
| Smoking status |  |  |  |  |  |  |  |  |  |  |
| Non-smoker | 147(21.9) | 168(24.4) | 115(18.9) | 106(20.1) | 0.225 | 325(93.1) | 209(92.5) | 253(95.1) | 231(95.1) | 0.195 |
| Ex-smoker | 325(48.5) | 330(47.8) | 321(52.7) | 277(52.6) |  | 10( 2.9) | 13( 3.9) | 10( 3.8) | 3( 1.2) |  |
| Current smoker | 198(29.6) | 192(27.8) | 173(28.4) | 144(27.3) |  | 14( 4.0) | 12( 3.6) | 3( 1.3) | 8( 3.3) |  |
| Missing | 0( 0.0) | 0( 0.0) | 0( 0.0) | 0( 0.0) |  | 0( 0.0) | 0( 0.0) | 0( 0.0) | 1( 0.4) |  |
| Alcohol consumption |  |  |  |  |  |  |  |  |  |  |
| Non-drinker | 107(16.0) | 119(17.3) | 95(15.6) | 94(17.8) | 0.863 | 203(58.2) | 202(60.5) | 178(66.9) | 156(64.2) | 0.407 |
| Ex-drinker | 88(13.1) | 81(11.7) | 77(12.6) | 58(11.0) |  | 23( 6.6) | 19( 5.7) | 15( 5.6) | 13( 5.4) |  |
| Current drinker | 474(70.8) | 490(71.0) | 437(71.8) | 373(70.8) |  | 123(35.2) | 113(33.8) | 73(27.4) | 73(30.0) |  |
| Missing | 1( 0.2) | 0( 0.0) | 0( 0.0) | 2( 0.4) |  | 0( 0.0) | 0( 0.0) | 0( 0.0) | 1( 0.4) |  |
| Regular exercise |  |  |  |  |  |  |  |  |  |  |
| No | 364(54.3) | 350(50.7) | 298(46.7) | 182(34.5) | <0.001 | 229(65.6) | 186(55.7) | 116(43.6) | 97(39.9) | <0.001 |
| Yes | 299(44.6) | 337(48.8) | 328(53.9) | 345(65.5) |  | 119(34.1) | 147(44.0) | 149(56.0) | 146(60.1) |  |
| Missing | 7( 1.0) | 3( 0.4) | 3( 0.5) | 0( 0.0) |  | 1( 0.3) | 1( 0.3) | 1( 0.4) | 0( 0.0) |  |
| Family history of cancer |  |  |  |  |  |  |  |  |  |  |
| No | 390(58.2) | 399(57.8) | 337(55.3) | 281(53.3) | 0.275 | 173(49.6) | 181(54.2) | 132(49.6) | 131(53.9) | 0.520 |
| Yes | 276(41.2) | 290(42.0) | 268(44.0) | 245(46.5) |  | 173(49.6) | 152(45.5) | 131(49.3) | 109(44.9) |  |
| Missing | 4( 0.6) | 1( 0.1) | 4( 0.7) | 1( 0.2) |  | 3( 0.9) | 1( 0.3) | 3( 1.1) | 3( 1.2) |  |
| Family history of colorectal cancer | |  |  |  |  |  |  |  |  |  |
| No | 625(93.3) | 639(92.6) | 574(94.3) | 489(92.8) | 0.406 | 320(91.7) | 307(91.9) | 245(92.1) | 221(91.0) | 0.966 |
| Yes | 41( 6.1) | 50( 7.3) | 31( 5.1) | 37( 7.0) |  | 26( 7.5) | 26( 7.8) | 18( 6.7) | 19( 7.8) |  |
| Missing | 4( 0.6) | 1( 0.1) | 4( 0.7) | 1( 0.2) |  | 3( 0.9) | 1( 0.3) | 3( 1.1) | 3( 1.2) |  |
| Calcium supplement intake | |  |  |  |  |  |  |  |  |  |
| No | 658(98.2) | 675(97.8) | 590(96.9) | 505(95.8) | 0.057 | 310(88.8) | 294(88.0) | 221(83.1) | 194(79.8) | 0.006 |
| Yes | 12( 1.8) | 15( 2.2) | 19( 3.1) | 22( 4.2) |  | 39( 11.2) | 40( 12.0) | 45( 16.9) | 49( 20.2) |  |

^a)^P-values are calculated by chi square test

Additional file 1: Table S3. Odds ratios (OR) and 95% confidence intervals (CI) for the association of dietary calcium intake and colorectal cancer risk among the calcium supplementation non-users

|  | Male (n=2,428) | | |  | Female (n=1,019) | | |
| --- | --- | --- | --- | --- | --- | --- | --- |
|  | Controls  /cases(n) | Age-adjusted OR(95%CI) | Multivariate  OR^a)^(95%CI) |  | Controls  /cases(n) | Age-adjusted OR(95%CI) | Multivariate  OR(95%CI) |
| Calcium intake (mg/day) |  |  |  | Calcium intake (mg/day) |  |  |  |
| Q1 (< 335) | 456/202 | 1.00 | 1.00 | Q1 (< 380) | 189/121 | 1.00 | 1.00 |
| Q2 (335 -< 432) | 454/221 | 1.09(0.87-1.38) | 0.92(0.71-1.19) | Q2 (380 -< 519) | 187/107 | 0.90(0.64-1.25) | 0.93(0.64-1.35) |
| Q3 (432 -< 567) | 450/140 | 0.68(0.53-0.88) | 0.51(0.38-0.68) | Q3 (519 -< 663) | 178/43 | 0.37(0.25-0.56) | 0.42(0.27-0.66) |
| Q4 (≥ 567) | 447/58 | 0.28(0.20-0.39) | 0.16(0.11-0.24) | Q4 (≥ 663) | 175/19 | 0.17(0.10-0.28) | 0.17(0.09-0.31) |
| P-value for trend^b)^ |  | <0.001 | <0.001 | P-value for trend |  | <0.001 | <0.001 |
| Dairy food calcium (mg/day) |  |  |  | Dairy food calcium (mg/day) |  |  |  |
| Q1 (<11) | 449/227 | 1.00 | 1.00 | Q1 (<20) | 187/105 | 1.00 | 1.00 |
| Q2 (11-<47) | 453/220 | 0.96(0.77-1.21) | 1.01(0.79-1.29) | Q2 (20-<78) | 185/99 | 0.95(0.67-1.33) | 1.05(0.71-1.54) |
| Q3 (47-<146) | 454/125 | 0.55(0.42-0.70) | 0.65(0.49-0.85) | Q3 (78-<225) | 178/63 | 0.63(0.44-0.92) | 0.72(0.47-1.09) |
| Q4 (≥146) | 451/49 | 0.21(0.15-0.29) | 0.28(0.19-0.39) | Q4 (≥225) | 179/23 | 0.23(0.14-0.37) | 0.22(0.13-0.38) |
| P-value for trend |  | <.001 | <.001 | P-value for trend |  | <0.001 | <0.001 |
| Non-Dairy food calcium  (mg/day) |  |  |  | Non-Dairy food calcium  (mg/day) |  |  |  |
| Q1 (<279) | 452/168 | 1.00 | 1.00 | Q1 (<302) | 184/109 | 1.00 | 1.00 |
| Q2 (279-<360) | 456/228 | 1.30(1.02-1.66) | 1.06(0.81-1.38) | Q2 (302-<397) | 179/104 | 0.96(0.68-1.35) | 0.84(0.57-1.23) |
| Q3 (360-<470) | 454/159 | 0.90(0.70-1.16) | 0.60(0.44-0.80) | Q3 (397-<522) | 185/57 | 0.51(0.35-0.74) | 0.54(0.35-0.83) |
| Q4 (≥470) | 445/66 | 0.37(0.27-0.50) | 0.16(0.11-0.24) | Q4 (≥522) | 181/20 | 0.18(0.11-0.30) | 0.15(0.08-0.28) |
| P-value for trend |  | <0.001 | <0.001 | P-value for trend |  | <0.001 | <0.001 |

^a)^ Adjusted by age, education level, regular exercise, fiber intake, calcium supplement status, and total energy intake

^b)^ Test for trend calculated with the median intake for each category of dietary calcium intake as a continuous variable.
